# The coexistence of myosteatosis and the creatinine/cystatin C ratio are determinants of outcomes in cholangiocarcinoma patients undergoing curative surgery

**DOI:** 10.3389/fonc.2024.1233768

**Published:** 2024-04-19

**Authors:** Yan Liu, Jingli Zhang, Guanghui Song, Xueli Ding, Hui Sun, Jianrui Zhou, Xue Jing

**Affiliations:** ^1^ Gastroenterology Department, The Affiliated Hospital of Qingdao University, Qingdao, Shandong, China; ^2^ Radiology Department, The Affiliated Hospital of Qingdao University, Qingdao, Shandong, China; ^3^ Inspection Department, The Affiliated Hospital of Qingdao University, Qingdao, Shandong, China

**Keywords:** cholangiocarcinoma, myosteatosis, creatinine/cystatin c ratio, radical excision, prognosis

## Abstract

**Background:**

Myosteatosis is a well-established predictor of poor prognosis in many types of cancer, and a decreased Creatinine/Cystatin C ratio (CCR) is a known indicator of unfavorable outcomes in patients with metabolic disorders and cancer. Despite this knowledge, the significance of concurrent CCR and myosteatosis in predicting the prognosis of patients with cholangiocarcinoma (CCA) who undergo radical surgery remains uncertain.

**Method:**

Data from 757 patients with cholangiocarcinoma who underwent the first radical resection in the Affiliated Hospital of Qingdao University from January 2017 to March 2022 were collected. According to the inclusion and exclusion criteria, 149 patients were finally included in the retrospective study cohort. Various clinicopathological, serological, and radiological data were collected at admission. Myosteatosis was evaluated using sliceOmatic software on computed tomography (CT) images. The study used receiver operating characteristic (ROC) curve analysis to determine the critical value of CCR, which predicts overall survival (OS) based on the Kaplan-Meier method. Univariate and multivariate Cox regression analyses were employed to identify the risk factors associated with OS and RFS confidently.

**Results:**

The group identified as the myosteatosis cohort consisted of 79 patients with an average age of 64.3 ± 7.8 years. The ROC curve analysis revealed an optimal critical CCR value of 10.834. A low CCR ≤ 10.834 and myosteatosis were found to be associated with poor OS and RFS outcomes (P = 0.022; P = 0.017; P = 0.038; P = 0.030 respectively). Moreover, patients with myosteatosis and a CCR ≤ 10.834 had the worst OS and RFS outcomes (P = 0.035; P = 0.027).

**Conclusion:**

After radical excision in CCA patients, the presence of myosteatosis and CCR had a negative correlation with prognosis. A more accurate prediction of OS and RFS was possible by combining CCR and myosteatosis, compared to CCR alone.

## Highlight

- Myosteatosis and CCR ≤ 10.834 strongly indicate survival in cholangiocarcinoma patients after surgery.

## 1 Introduction

Cholangiocarcinoma (CCA) is a significant hepatic malignancy with a grim prognosis even after radical excision (1–3). The need for novel and readily measurable prognostic markers is paramount in the quest for personalized medicine and precision therapies (4, 5).

Myosteatosis is characterized by the infiltration of adipose and connective tissue into the intermuscular milieu, leading to declining muscle mass (6). Myosteatosis can be evaluated by examining the average degeneration of the skeletal muscle area (SMA) (7, 8). SMA degeneration reliably predicts disease-related complications and overall survival (OS) (9). Patients with CCA often suffer from heightened caloric consumption, decreased liver functionality, jaundice, and protein deficiency, leading to malnutrition, metabolic disorders, a drastic reduction in muscle mass, the onset of myosteatosis, and other alterations in body composition.

Creatinine is a substance that is produced when muscles break down. It is a dependable measure of the total muscle mass present in the body (10, 11). In contrast, Cystatin C, an indicator of the kidney’s glomerular filtration rate, emanates from all nucleated cells in the body at an unwavering rate, and its plasma concentration remains unimpacted by muscle mass (12). A diminished Creatinine/Cystatin C ratio (CCR) has been correlated with unfavorable prognoses in gastric cancer patients (13). However, the correlation between CCR and prognosis in the cohort of patients suffering from CCA remains largely unexplored.

Therefore, this investigation aims to determine how accurately the combination of CCR and myosteatosis predicts the prognosis of CCA patients after radical surgery. The findings of this study could provide valuable insights into the prognostic markers of CCA, leading to more accurate prognostic estimates and better-informed treatment decisions.

## 2 Materials and methods

### 2.1 Patients

A retrospective analysis was conducted on patients with cholangiocarcinoma (CCA) who underwent radical surgical excision at the Affiliated Hospital of Qingdao University between January 2017 and January 2022—the follow-up period extended until December 31, 2022. The selection criteria included: 1) Patients who underwent radical resection and were confirmed to have CCA through pathology after surgery; 2) Patients who underwent preoperative imaging with computed tomography (CT) that included imaging of the third lumbar vertebra level of the spine, with images suitable for body composition analysis; 3) Patients who had a complete set of clinicopathological and serological data collected within a month before surgery, including creatinine and cystatin C levels; 4) Patients with access to follow-up data and informed consent. Patients who received local and systemic adjuvant chemoradiotherapy before radical resection, those with concurrent tumors, and those unable to complete all follow-up assessments were excluded. The Ethics Committee of the Affiliated Hospital of Qingdao University approved this study, waiving the requirement for informed consent due to the retrospective study design and analysis of existing clinical data. (QYFYWZLL27646)

### 2.2 Collection of clinicopathologic data

Extensive data was collected from the electronic medical record system of the Affiliated Hospital of Qingdao University. Patient demographic and serological data were obtained and recorded a month before surgery. The demographic data included age, sex, weight, and height. Serological data included assessments of renal function, cystatin C and gamma-glutamyl transferase levels, and the measurement of liver function markers such as alanine transaminase (ALT), aspartate transaminase (AST), albumin (ALB), globulin (GLB), total bilirubin (TBIL), and direct bilirubin (DBIL).

The details of the surgery and pathology were collected, including the location, diameter, and degree of differentiation of the tumor. The Creatinine/Cystatin C ratio (CCR) was calculated using the following formula: CCR = [creatinine (mg/dL)/cystatin C (mg/L)] × 10 (14).

The primary focus of the study was to measure the overall survival (OS) of the participants. The duration between the surgery date and the date of death or the end of the follow-up period was considered for calculating OS. The secondary endpoint was recurrence-free survival (RFS), defined as the period from the surgery date to the first recurrence or death. Recurrence was determined by either imaging-based or pathological validation of a tumor.

### 2.3 Body composition analysis based on CT

CT imaging data of patients collected within a month before surgery were analyzed using the sliceOmatic^®^ (v5.0; TomoVision, Canada) software. The software was used to quantify the skeletal muscle and adipose tissue content at the third lumbar vertebra level to evaluate sarcopenia and myosteatosis according to previously established parameters (15). Different specific Hounsfield unit (HU) thresholds were assigned for various tissues: -29 to +150 HU for skeletal muscle, -150 to -50 HU for visceral adipose tissue, and -190 to -30 HU for subcutaneous and intramuscular adipose tissue (16).

**Figure 1:**
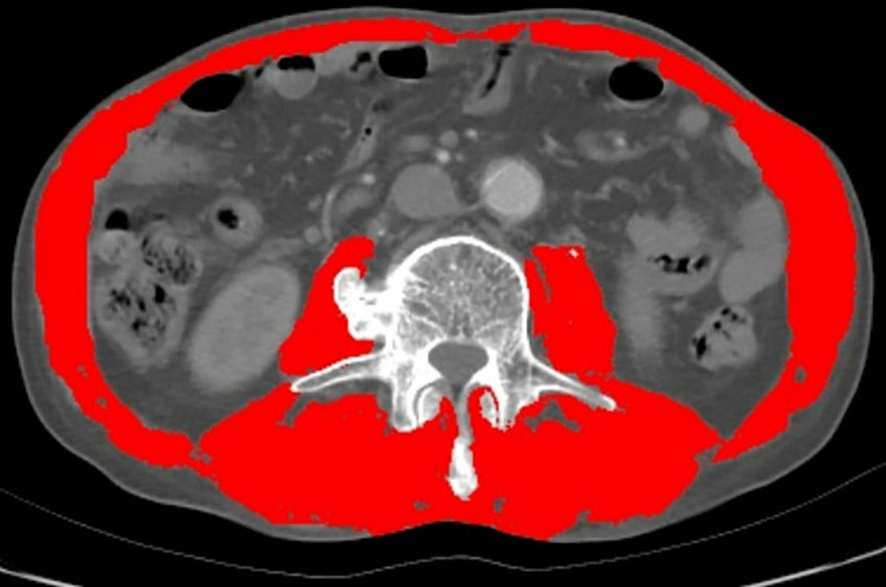
Evaluation of muscle quantity and quality at the third lumbar vertebra of the spine by computed tomography (CT). (The red part).

Each tissue type at the L3 level was evaluated in every CT image and marked with different colors (**Figure 1**). Sarcopenia was assessed through the skeletal muscle index (SMI), calculated as [SMA/height squared (m²)], while myosteatosis was assessed by the average SMA degeneration (17). The myosteatosis thresholds were set at 41 HU for patients with a body mass index (BMI) < 25 kg/m² and 33 HU for patients with a BMI ≥ 25 kg/m² (18). Sarcopenia was defined as an SMI of < 41 cm²/m² for women and < 43 cm²/m² for men with a BMI < 25 and < 53 cm²/m² for men with a BMI ≥ 25 (19).

All measurements were carried out by a pair of researchers who analyzed the CT images of all patients blindly and with great attention to detail.

### 2.4 Statistical analysis

Using specialized software, measurement data, normally distributed data, and data with homogeneous variance underwent statistical testing. Non-parametric tests were employed for alternative data types. Depending on the type of data (parametric and non-parametric), the data are represented as either the mean ± standard deviation (SD) or the median (P25–P75). Fractional variables are represented as frequency and percentage.

Two-sample t-tests or Wilcoxon Mann-Whitney (U) tests were employed to compare numerical data. In contrast, categorical variables between the two groups were compared using Pearson Chi-square tests or Fisher exact tests. A receiver operating characteristic (ROC) curve of the CCR was drafted to calculate the critical value for predicting the survival status of patients with CCA.

Based on this cut-off value, all patients were grouped into either a low CCR group or a high CCR group. Kaplan-Meier curve analysis was conducted to evaluate OS and RFS, and the log-rank test was used to examine the differences between curves. Moreover, univariate and multivariate Cox regression analyses were conducted for OS and RFS, respectively, to analyze the prognostic value of the variables for OS and RFS based on the hazard ratio (HR) and 95% confidence interval (CI 95%).

All statistical analyses were conducted using the Statistical Package for the Social Sciences (SPSS) (version 26.0; IBM, Armonk, NY, USA). A two-sided P-value of less than 0.05 was considered statistically significant.

## 3 Results

### 3.1 Baseline features

**Figure 2:**
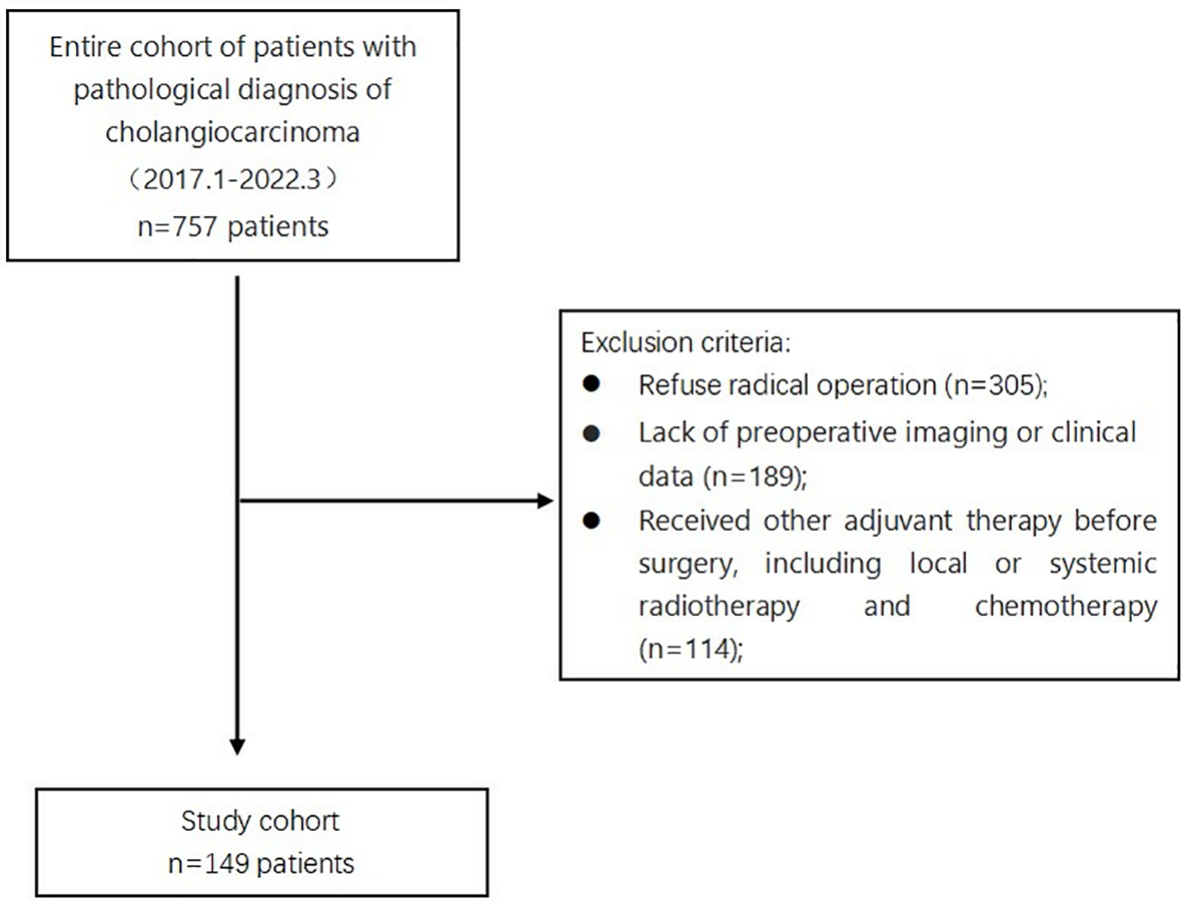
Flow diagram of patient inclusion/exclusion criteria.

Among the 757 patients pathologically diagnosed with CCA, 608 were excluded based on specific criteria. The study focused on 149 patients diagnosed with cholangiocarcinoma (CCA). They were separated into two groups - one with myosteatosis and one without. The research flow chart is shown in **Figure 2**.

#### 3.1.1 Clinicopathological features and demographic data

Among 149 patients, 79 were in the myosteatosis group, and 70 were in the non-myosteatosis group according to diagnostic criteria. The study found no significant difference in age between the two groups, but there were differences in gender and lifestyle factors. The non-myosteatosis group had a higher percentage of smokers and alcohol consumers than the myosteatosis group. The demographic and pathological baseline data of the patients are shown in **Table 1.1**.

**Table 1.1:** Demographic and pathological baseline data.

Factors	Myosteatosis N = 79	Non-myosteatosis N = 70	P
Age (years)	64.3 ± 7.8	58.0 ± 9.9	0.118
Gender Male Female	32 (40.5) 47 (59.5)	53(75.7) 17(24.3)	**0.000**
BMI(kg/m²) <25kg/m² ≥25kg/m²	59 (74.7) 20 (25.3)	30(42.9) 40(57.1)	**0.000**
Smoke	14 (17.7)	26(37.1)	**0.008**
Drink	13 (16.5)	21(30)	0.049
Hypertension	23 (29.1)	16(22.9)	0.386
Diabetes	17 (21.5)	7(10)	0.056
Hepatitis B	8 (10.1)	10(14.3)	0.437
Liver cirrhosis, n (%)	9 (11.4)	13(18.6)	0.218
Tumor size, n(%) ≥ 5 cm <5 cm	40 (50.6) 39 (49.4)	27(38.6) 43(61.4)	0.140
Tumor differentiation, n (%) low median-low median high	21 (26.6) 19 (24.1) 37 (46.8) 2 (2.5)	17(24.3) 23(32.9) 29 (41.4) 1(1.4)	0.653
Nerve invasion, n (%)	37 (46.8)	33(47.1)	0.970
Vascular invasion, n (%)	25 (31.6)	26(37.1)	0.480
Lymph node metastasis, n (%)	22 (27.8)	16(22.9)	0.485
Biliary calculus, n (%)	17 (78.5)	11(15.7)	0.365
Recurrence Yes No	22(27.8%) 57(72.2%)	15(21.4%) 55(78.6%)	0.365

Bolded values mean: P > 0.05.

**Table 1.2:** Serological baseline data.

Factors	Myosteatosis N = 79	Non-myosteatosis N = 70	P
ALB	37.9 ± 5.9	41.0 ± 5.2	0.166
**GLB**	28.0 ± 5.4	26.6 ± 4.0	**0.010**
**CA 125**	18.3 (10.5-41.4)	13.7 (9.9-21.7)	**0.029**
CEA	3.4 (2.2-6.4)	2.9(1.8-5.4)	0.154
AFP	3.3 (2.3-4.8)	2.9 (2.2-4.6)	0.291
ALT	25.0 (17.0-67.4)	26.5(16.0-64.9)	0.746
AST	29.0 (20.0-55.0)	23.0 (18.0-37.0)	0.161
WBC	6.3 (5.2-8.0)	6.7 (5.5-7.6)	0.953
Neutrophil	3.8 (3.1-5.2)	4.1 (3.2-5.1)	0.882
Lymphocyte	1.7 ± 0.5	1.8 ± 0.6	0.417
Monocyte	0.5 (0.4-0.6)	0.5 (0.4-0.6)	0.663
Thrombocyte	239.1 ± 80.5	229.0 ± 79.7	0.345
HB	127.0 ± 18.5	137.9 ± 18.6	0.649
TAIL	16.0 (11.0-42.5)	17.1(12.6-34.4)	0.535
DBIL	5.7 (3.9-32.1)	4.8 (3.4-15.0)	0.372
**IBIL**	10.0 (6.5-16.0)	12.0(8.7-20.0)	**0.032**
**Alkaline phosphatase**	111.0 (81.3-236.0)	87.5(67.0-187.8)	**0.042**
Ureophil	5.4 ± 1.7	5.8 ± 1.7	0.706
**Uric Acid**	271.0 ± 105.1	304.8 ± 105.5	**0.037**
Creatinine	59.5 ± 20.0	71.9 ± 22.5	0.691
PT	11.0 ± 1.6	11.0 ± 1.5	0.517
Fibrinogen	3.5 (3.0-4.5)	3.3 (2.8-4.0)	0.131
APTT	31.2 (28.4-34.0)	30.6 (28.1-32.8)	0.644
APTT ratio	1.1 ± 0.1	1.1 ± 0.2	0.371
TT	15.8 ± 2.5	16.3 ± 2.1	0.767
TTratio	1.11 (1.0-1.2)	1.1 (1.0-1.2)	0.581
AT III	93.2 (79.4-109.0)	97.0 (86.0-114.1)	0.390
D-dimer	400.0 (230.0-650.0)	330.0 (238.0-440.0)	0.071
PTratio	1.0 ± 0.1	1.0 ± 0.1	0.365
**CCR**	6.79 (5.78-9.23)	8.09 (6.77-10.80)	**0.001**

ALB, albumin; GLB, globulin; CA125, carbohydrate antigen 125; CEA, carcinoembryonic antigen; AFP, alpha-fetoprotein; ALT, alanine transaminase; AST, aspartate transaminase; WBC, white blood cell; HB, hemoglobin; TBIL, total bilirubin; DBIL, direct bilirubin; IBIL, indirect bilirubin; CCR, creatinine/cystatin C ratio.

Bolded values mean: P > 0.05.

**Table 1.3:** Body composition characteristics.

Factors	Myosteatosis N = 79	Non-myosteatosis N = 70	P
SMI, cm²	41.92 ± 6.85	49.73 ± 8.55	0.141
SMA, HU	113.66 ± 23.81	140.98 ± 29.39	0.085
SAT, cm²	141.21 ± 63.03	132.17 ± 63.50	0.803
VAT, cm²	108.10 (65.15-169.05)	129.80 (74.75-217.88)	0.138
**IMAT**, cm²	7.82 ± 5.02	5.82 ± 3.68	**0.019**
**VSR**	0.72 (0.47-1.10)	1.05 (0.69-1.27)	**0.008**
**Sarcopenia** Yes No	45 (57%) 34 (43%)	26 (37.1%) 44 (62.9%)	**0.016**

SMI stands for skeletal muscle index; SMA stands for skeletal muscle attenuation. SAT stands for subcutaneous adipose tissue, VAT stands for visceral adipose tissue, IMAT stands for intermuscular adipose tissue, and VSR stands for visceral-to-subcutaneous adipose tissue ratio. Bolded values mean: P > 0.05.

#### 3.1.2 Serological baseline data

The study examined the patients’ serological characteristics (**Table 1.2**). The myosteatosis group showed higher levels of certain substances, such as GLB, CA-125, and alkaline phosphatase, compared to the non-myosteatosis group. On the other hand, uric acid levels and CCR were lower for the myosteatosis group.

#### 3.1.3 Body composition characteristics data

The study also examined the characteristics of the patient’s body composition (**Table 1.3**). It found a higher proportion of individuals with sarcopenia and a significantly higher quantity of intermuscular adipose tissue (IMAT) in the myosteatosis group. The myosteatosis group also had a significantly lower visceral to subcutaneous fat ratio (VSR).

#### 3.1.4 Determination of the optimal critical value of CCR

**Figure 3:**
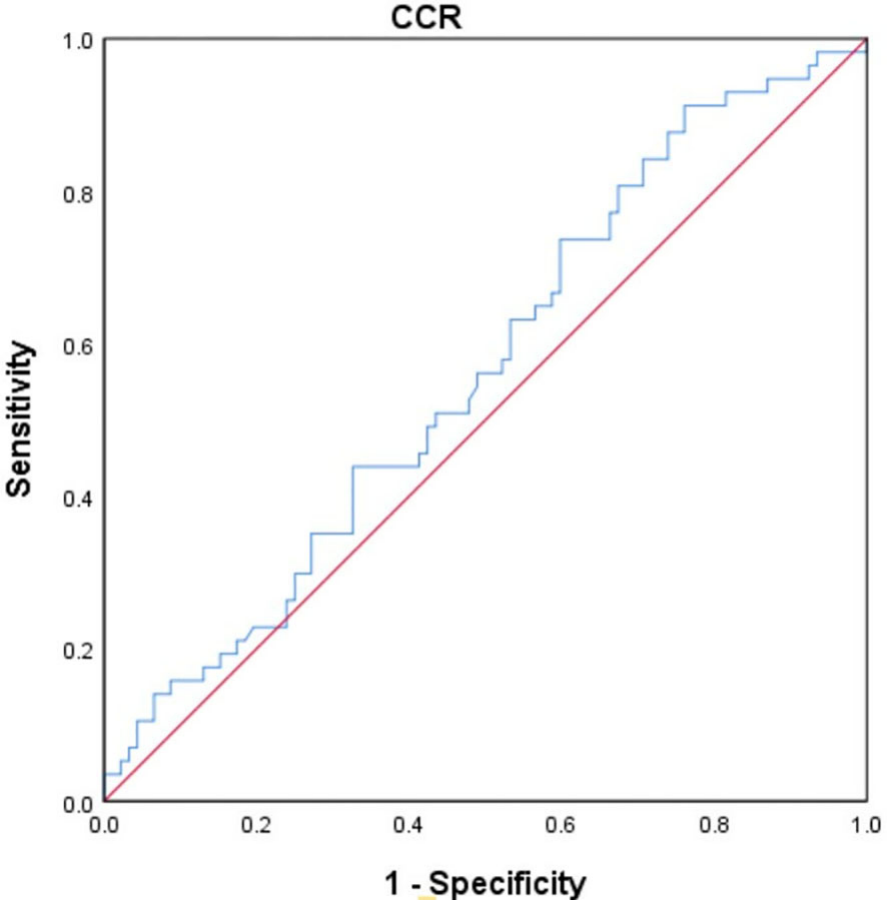
The ROC curve was generated to evaluate the discriminatory ability of the CCR. ROC, receiver operating characteristic curve; CCR, creatinine/cystatin C ratio.

The study utilized the receiver operating characteristic (ROC) curve to determine the optimal cut-off value for CCR in predicting overall survival. (OS) (**Figure 3**). The best truncation of the CCR was 10.834, meaning patients with a CCR above 10.834 could potentially have better OS than those with a lower CCR. However, it has been noted that the CCR may not be a robust standalone predictor for OS in patients with cholangiocarcinoma.

### 3.2 The OS and RFS of intrahepatic and extrahepatic cholangiocarcinoma

The study classified the patients according to anatomic location, resulting in 80 intrahepatic cholangiocarcinomas and 69 extrahepatic cholangiocarcinomas. Patients with extrahepatic cholangiocarcinoma had significantly shorter OS and RFS than those with intrahepatic cholangiocarcinoma (**Supplementary Figure S1**).

### 3.3 Univariate and multivariate Cox regression analysis of overall survival

The study identified different factors that affect the survival rate of patients with cholangiocarcinoma. One of the factors is myosteatosis, which is when fat accumulates in muscle tissue. Patients with myosteatosis have almost twice the chance of not surviving compared to those without it. Another factor is the creatinine/cystatin C ratio (CCR). If the ratio is less than or equal to 10.834, it may lead to a lower survival rate. Lymph node involvement is also a significant factor for worse survival, while low albumin levels show poor nutritional status. Higher CA-125 levels may indicate cancer progression, while elevated white blood cell (WBC), neutrophil, and monocyte counts show systemic inflammation that may impact survival. Lower hemoglobin levels may indicate anemia from chronic disease, and higher levels of total bilirubin, direct bilirubin, indirect bilirubin, alkaline phosphatase, and uric acid may indicate impaired liver function and metabolic disturbances. Among all the variables, only lymph node metastasis showed a significant correlation with OS (Hazard Ratio [HR] = 3.126, P < 0.0001). Patients with lymph node metastasis had a 3.1 times higher chance of having a worse prognosis (**Table 2**).

**Table 2:** Univariate and multivariate Cox proportional hazards regression analysis of OS.

Variables	Univariate analysis	Multivariate analysis
	HR (95% CI) P	HR (95% CI) P
Age	1.029 (0.999-1.059) 0.057	
Gender	1.220 (0.725-2.051) 0.454	
Tumor size	0.665 (0.394-1.121) 0.125	
Nerve invasion	0.692 (0.411-1.165) 0.166	
Vascular invasion	0.602 (0.357-1.017) 0.058	
Lymph node metastasis	3.435 (2.030-5.810) **0.000**	3.126 (1.758-5.559) **0.000**
Biliary calculus	0.781 (0.413-1.478) 0.448	
ALB	0.930 (0.890-0.972) **0.001**	0.973 (0.906-1.046) 0.458
GLB	1.040 (0.986-1.098) 0.150	
CA125	1.002 (1.001-1.003) **0.004**	1.001 (1.000-1.003) 0.059
WBC	1.193 (1.105-1.287) **0.000**	1.122 (0.616-2.043) 0.708
Neutrophil	1.218 (1.125-1.318) **0.000**	1.186 (0.635-2.215) 0.593
Lymphocyte	0.751 (0.459-1.229) 0.255	
Monocyte	3.869 (1.342-11.154) **0.012**	0.238 (0.017-3.296) 0.285
Thrombocyte	1.002 (0.998-1.005) 0.369	
HB	0.981 (0.968-0.993) **0.002**	1.004 (0.986-1.023) 0.634
TBIL	1.002 (1.000-1.004) **0.024**	1.011 (0.979-1.044) 0.493
DBIL	1.003 (1.000-1.005) **0.025**	0.987 (0.944-1.032) 0.570
IBIL	1.007 (1.000-1.013) **0.035**	NA
Alkaline phosphatase	1.001 (1.000-1.003) **0.019**	1.000 (0.998-1.001) 0.639
Ureophil	0.914 (0.783-1.068) 0.256	
Uric Acid	0.997 (0.994-1.000) **0.033**	1.000 (0.996-1.003) 0.865
Creatinine	0.992 (0.980-1.005) 0.211	
CCR	2.547 (0.017-6.383) **0.046**	2.654 (0.880-8.004) 0.083
Recurrence	0.879 (0.498-1.551) 0.655	
Myosteatosis	1.882 (1.084-3.268) **0.025**	1.779 (0.938-3.372) 0.078
Sarcopenia	0.935 (0.556-1.572) 0.798	

BMI, body mass index; ALB, albumin; GLB, globulin; CA125, carbohydrate antigen 125; CEA, carcinoembryonic antigen; AFP, alpha-fetoprotein; ALT, alanine transaminase; AST, aspartate transaminase; WBC, white blood cell; HB, hemoglobin; TBIL, total bilirubin; DBIL, direct bilirubin; IBIL, indirect bilirubin; CCR, creatinine/cystatin C ratio; SMI, skeletal muscle index; SMA, skeletal muscle attenuation; SAT, subcutaneous adipose tissue; VAT, visceral adipose tissue; IMAT, intermuscular adipose tissue; VSR, visceral-to-subcutaneous adipose tissue ratio.

Bolded values mean: P > 0.05.

However, age, body mass index (BMI), nerve invasion, vascular invasion, skeletal muscle area (SMA), subcutaneous adipose tissue (SAT), visceral adipose tissue (VAT), intermuscular adipose tissue (IMAT), visceral to subcutaneous fat ratio (VSR), and sarcopenia did not affect the overall survival (OS) of individuals with cholangiocarcinoma (**Supplementary Table S1**).

### 3.4 Univariate and multivariate Cox regression analysis of relapse-free survival

The research showed that certain factors were linked to cholangiocarcinoma recurrence in patients. These included the presence of myosteatosis, a CCR score of 10.834 or less, and various laboratory parameters such as alkaline phosphatase, hemoglobin, and cancer antigen 125. Only three factors independently influenced patients’ recurrence-free survival (RFS): lymph node metastasis, vascular invasion, and cancer antigen 125 levels. These three factors significantly predict recurrence or death, even when accounting for other variables (**Table 3**).

**Table 3:** Univariate and multivariate Cox proportional hazards regression analysis of RFS.

Variables	Univariate analysis	Multivariate analysis
	HR (95% CI) P	HR (95% CI) P
Tumor size	1.687 (1.001-2.842) **0.050**	1.639 (0.872-3.080) 0.125
Nerve invasion	0.699 (0.415-1.176) 0.177	
Vascular invasion	1.770 (1.048-2.988) **0.033**	1.989 (1.104-3.582) **0.022**
Lymph node metastasis	3.411 (2.011-5.786) **0.000**	2.947 (1.638-5.301) **0.000**
Biliary calculus	0.791 (0.418-1.497) 0.472	
ALB	0.926 (0.886-0.967) **0.001**	0.962 (0.892-1.038) 0.315
GLB	1.050 (0.993-1.109) 0.085	
CA125	1.002 (1.001-1.003) **0.004**	1.001 (1.000-1.003) **0.037**
WBC	1.186 (1.103-1.274) **0.000**	1.199 (0.660-2.177) 0.551
Neutrophil	1.211 (1.123-1.305) **0.000**	1.064 (0.569-1.990) 0.847
Lymphocyte	0.780 (0.482-1.262) 0.311	
Monocyte	4.956 (1.732-14.180) **0.003**	0.318 (0.020-4.945) 0.413
Thrombocyte	1.002 (0.999-1.006) 0.225	
HB	0.979 (0.967-0.991) **0.001**	1.006 (0.987-1.025) 0.561
TBIL	1.002 (1.000-1.003) **0.049**	1.020 (0.985-1.055) 0.269
DBIL	1.002 (1.000-1.005) **0.045**	0.976 (0.931-1.024) 0.326
IBIL	1.006 (1.000-1.012) 0.069	
Alkaline phosphatase	1.001 (1.000-1.002) **0.022**	1.000 (0.998-1.002) 0.982
Ureophil	0.895 (0.767-1.043) 0.155	
Uric Acid	0.997 (0.995-1.000) **0.040**	1.001 (0.997-1.004) 0.708
Creatinine	0.992 (0.980-1.005) 0.217	
**CCR**	2.648 (1.057-6.633) **0.038**	2.520 (0.837-7.591) 0.100
Recurrence	0.588 (0.332-1.042) 0.069	
**Myosteatosis**	1.931 (1.113-3.352) **0.019**	1.751 (0.914-3.354) 0.091
Sarcopenia	0.906 (0.539-1.523) 0.709	

BMI, body mass index; ALB, albumin; GLB, globulin; CA125, carbohydrate antigen 125; CEA, carcinoembryonic antigen; AFP, alpha-fetoprotein; ALT, alanine transaminase; AST, aspartate transaminase; WBC, white blood cell; HB, hemoglobin; TBIL, total bilirubin; DBIL, direct bilirubin; IBIL, indirect bilirubin; CCR, creatinine/cystatin C ratio; SMI, skeletal muscle index; SMA, skeletal muscle attenuation; SAT, subcutaneous adipose tissue; VAT, visceral adipose tissue; IMAT, intermuscular adipose tissue; VSR, visceral-to-subcutaneous adipose tissue ratio.

Bolded values mean: P > 0.05.

Other factors, such as age, body mass index, nerve invasion, lymphocyte count, indirect bilirubin levels, and sarcopenia, were not found to have a significant impact (**Supplementary Table S2**).

### 3.5 The presence of myosteatosis is associated with shorter OS and RFS

**Figure 4:**
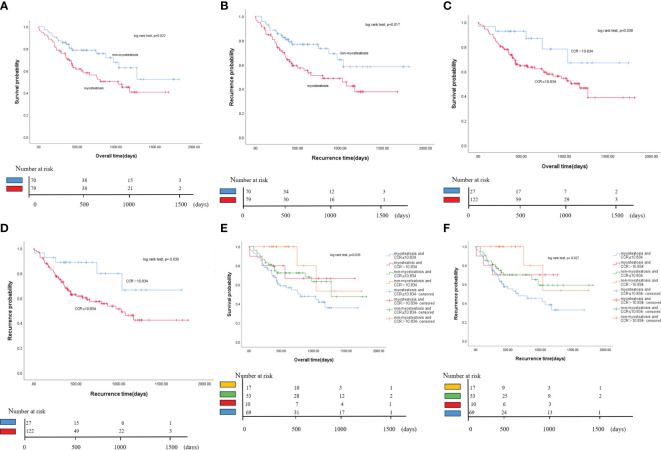
Kaplan-Meier curves of OS and RFS for all patients. **(A)** Comparison of OS between myosteatosis and non-myosteatosis patients. **(B)** Comparison of RFS between myosteatosis and non-myosteatosis patients. **(C)** Comparison of OS between patients with a CCR ≤ 10.834 and those with a CCR > 10.834. **(D)** Comparison of RFS between patients with a CCR ≤ 10.834 and those with a CCR > 10.834. **(E)** Kaplan-Meier curves of OS for all patient groups based on the presence or absence of myosteatosis and the CCR. **(F)** Kaplan-Meier curves of RFS for all patient groups based on the presence or absence of myosteatosis and the CCR. OS, overall survival; RFS, recurrence-free survival; CCR, creatinine/cystatin C ratio.

The study found that patients with myosteatosis had significantly shorter median survival than those without myosteatosis (33.4 months vs. 59.9 months, P = 0.022). It also found that myosteatosis was associated with a shorter median RFS than non-myosteatosis. This suggests that the presence of myosteatosis will increase the risk of disease recurrence or death in cholangiocarcinoma patients (**Figures 4A, B**).

### 3.6 CCR ≤ 10.834 is associated with shorter OS and RFS

The study found that if the ratio is less than or equal to 10.834, it may lead to a lower survival rate. Individuals with a creatinine/cystatin C ratio (CCR) of ≤ 10.834 had significantly shorter median survival than those with a CCR > 10.834 (37.2 months vs. 57.9 months, P = 0.038). Similarly, the study also found that a lower CCR score was associated with a shorter median RFS than a higher CCR score. This suggests that the presence of a lower CCR score increases the risk of disease recurrence or death in cholangiocarcinoma patients (**Figures 4C, D**).

### 3.7 Prognostic value of the combination of myosteatosis and the CCR ≤ 10.834

This study found that patients with cholangiocarcinoma may have a worse prognosis if they have both myosteatosis and a CCR score of 10.834 or less. Patients with these two factors had the lowest overall and recurrence-free survival rates. Patients without myosteatosis and a CCR score of more than 10.834 had the highest survival rate. In contrast, those with both myosteatosis and a CCR score of 10.834 or less had the lowest survival rate. The difference in overall survival between these two groups was 31.8 months. The median recurrence-free survival for the group with both myosteatosis and a CCR score of 10.834 or less was 20.8 months, significantly less than the other groups. These findings suggest that combining myosteatosis and a CCR score of 10.834 or less may help predict the prognosis of patients with cholangiocarcinoma (**Figures 4E, F**).

## 4 Discussion

Myosteatosis is a condition where fat builds up in muscles. It can be detected through CT scans at the level of the third lumbar vertebrae (20). Studies have shown that myosteatosis hurts the prognosis of various types of cancer, especially in malnourished patients (21, 22). In patients with cholangiocarcinoma (CCA), myosteatosis is common and can lead to malnutrition and cachexia. A study of 149 CCA patients found that over half of them (53.0%) developed myosteatosis (21). A meta-analysis has confirmed a significant decrease in survival rates for cancer patients who develop myosteatosis ^(^ 23 ^).^. This study shows that myosteatosis is associated with poor overall survival (OS) and recurrence-free survival (RFS) in patients with CCA who underwent radical resection.

Sarcopenia and myosteatosis are two conditions that can affect body composition. In certain types of cancer, sarcopenia has been linked to poor prognosis (24, 25). The scientific community has an ongoing discussion regarding the correlation in question. In the case of patients with cholangiocarcinoma, the study shows that while myosteatosis is associated with poor overall survival (OS) and recurrence-free survival (RFS), sarcopenia by itself does not show a significant correlation with survival rates (26).

Myosteatosis can be an early warning sign of disease progression. It can cause changes in muscle strength and functionality that can be detected before abnormal shifts occur (27). While the details are still unknown, some theories suggest that myosteatosis can lead to metabolic disorders and changes in nutrition, which can disrupt overall body health (28). Fat accumulation in the body leads to inflammation, which can contribute to the development and progression of various diseases, including cancer (29). Studies have shown that increased levels of inflammatory markers and cells are predictors of survival and recurrence-free survival in patients with cholangiocarcinoma (30, 31).

Cholangiocarcinoma can be divided into intrahepatic and extrahepatic cholangiocarcinoma according to anatomic location, and extrahepatic cholangiocarcinoma includes hilar cholangiocarcinoma and distal cholangiocarcinoma (32). The preoperative diagnosis of distal cholangiocarcinoma and pancreatic ductal adenocarcinoma has been a focus of research (33). Distal cholangiocarcinoma and pancreatic ductal adenocarcinoma are two types of highly aggressive cancers that are associated with an inferior prognosis (34). According to this study, myosteatosis is a medical condition where muscle tissue is replaced by fat. The research has shown that this condition is associated with a poorer prognosis in people with pancreatic ductal adenocarcinoma. The primary reason for this is that the tumor blocks the liver, leading to liver dysfunction. Furthermore, myosteatosis can trigger changes in muscle composition through the communication between the liver and muscle tissues (35, 36). These findings suggest that interventions that target myosteatosis could improve outcomes in Distal cholangiocarcinoma and pancreatic ductal adenocarcinoma and warrant further investigation.

Creatinine is a marker used to check the health of the kidneys. However, because the creatinine produced is linked to muscle mass, it may not be a reliable marker for muscle loss (37). Cystatin C is a better indicator of how well the kidneys filter blood because muscle mass does not affect it (38). Recent studies have shown that the creatinine-cystatin C ratio (CCR) may be linked to increased fat in muscles and decreased muscle mass, particularly in people who are not in good physical health (39–42).

Previous studies have shown that Creatinine/Cystatin C (CCR) is a substitute indicator for sarcopenia in patients with gastric cancer, gastrointestinal stromal tumors, and esophageal cancer (42–44). Because creatinine is affected by chronic disease consumption, poor nutritional status, and other pathological conditions, the creatinine level will decrease in cases of skeletal muscle atrophy. In contrast, cystatin C is not affected by muscle metabolism. In this study, researchers divided patients into four groups based on myosteatosis and CCR to further investigate how these factors affect prognosis. Due to the limited sample size of this study, the role of CCR in the development of sarcopenia in patients with cholangiocarcinoma cannot be analyzed at this time.

This study investigates the combined predictive capacity of myosteatosis and CCR. The results showed that patients with both conditions and a CCR of 10.834 or less had significantly worse overall survival and recurrence rates than other groups. These findings suggest that combining these measures could be of clinical significance in assessing the prognosis of CCA patients.

However, as a retrospective study, there is a risk of selection bias, so future prospective studies are needed to validate these findings. Additionally, the study’s single-center, case-control design may not provide a fully representative sample, so multicenter and prospective research must confirm these observations.

## 5 Conclusion

This study found that patients with CCA who had myosteatosis and a CCR of 10.834 or less are both associated with poorer OS and RFS in patients with CCA who underwent radical surgery. Notably, patients with both myosteatosis and a CCR of 10.834 or less presented the poorest OS and RFS among all groups studied.

In conclusion, this study emphasized the significance of preoperative evaluation of myosteatosis and CCR in creating better treatment plans and nutritional strategies for CCA patients. The findings provide valuable insights to oncologists and other medical practitioners in designing efficacious treatment plans and augmenting outcomes for this patient cohort.

## Data availability statement

The raw data supporting the conclusions of this article will be made available by the authors, without undue reservation.

## Ethics statement

The Ethics Committee of the Affiliated Hospital of Qingdao University (QYFYWZLL27646) bestowed approval on this study, waiving the requirement for informed consent in view of the retrospective study design and the analysis of existing clinical data. The studies were conducted in accordance with the local legislation and institutional requirements. The participants provided their written informed consent to participate in this study. Written informed consent was obtained from the individual(s) for the publication of any potentially identifiable images or data included in this article.

## Author contributions

Conceptualization, YL and XJ; methodology, HS and XJ; software, YL, XJ, and XD; validation, XD, XJ, and HS; formal analysis, HS, XJ, and JZ.; investigation, XD and GS; resources, YL, XJ, and GS; data curation, YL, GS, HS, and XJ; writing—original draft preparation, XD and XJ; writing—review and editing, HS, GS, and XJ; visualization, JZ, XD, and XJ; supervision, HS, XD, and XJ; project administration, XD, HS, and XJ; funding acquisition, XJ. All authors have read and agreed to the published version of the manuscript.
