# Supplementary material for: The coexistence of myosteatosis and the creatinine/cystatin C ratio are determinants of outcomes in cholangiocarcinoma patients undergoing curative surgery
Source: Front Oncol. 2024 Apr 19;14:1233768. doi: 10.3389/fonc.2024.1233768 (PMC11066224; doi:10.3389/fonc.2024.1233768)
Supplement: Supplementary file 1 [file DataSheet_1.docx]

Figure S1. Kaplan-Meier curves of OS and RFS for intrahepatic and extrahepatic cholangiocarcinoma. (a) Comparison of OS between intrahepatic and extrahepatic cholangiocarcinoma. (b) Comparison of RFS between intrahepatic and extrahepatic cholangiocarcinoma.

**（a）**


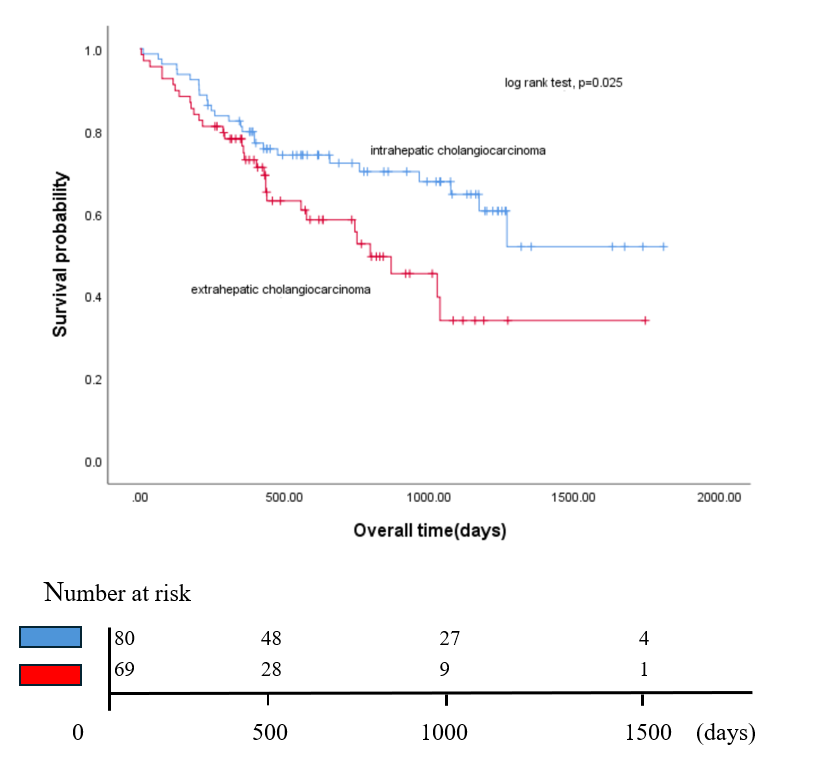


**（b）**


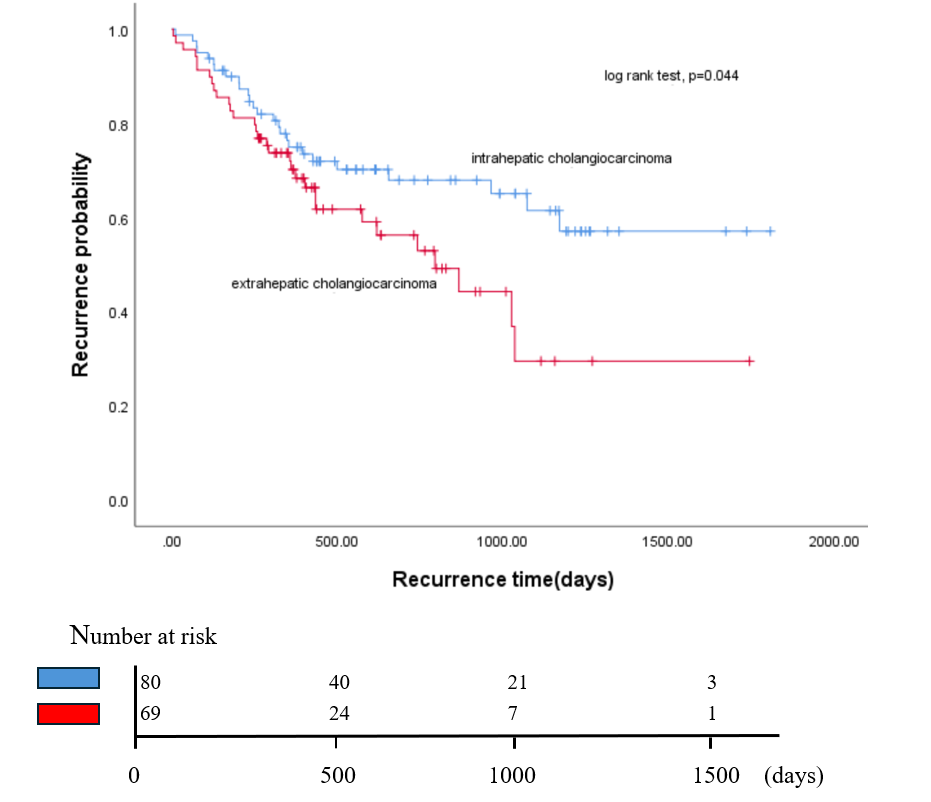


Table S1. Univariate and multivariate Cox proportional hazards regression analysis of OS.

| Variables | Univariate analysis | Multivariate analysis |
| --- | --- | --- |
|  | HR (95% CI) P | HR (95% CI) P |
| BMI | 0.842 (0.499-1.422) 0.520 |  |
| Hepatitis B | 1.561 (0.623-3.912) 0.342 |  |
| Liver cirrhosis | 2.037 (0.813-5.103) 0.129 |  |
| CEA | 1.000 (0.998-1.002) 0.936 |  |
| AFP | 0.997 (0.988-1.007) 0.610 |  |
| ALT | 1.001 (0.998-1.003) 0.584 |  |
| AST | 1.001 (0.998-1.004) 0.431 |  |
| SMI | 0.989 (0.959-1.020) 0.478 |  |
| SMA | 0.997 (0.988-1.005) 0.443 |  |
| SAT | 1.000 (0.996-1.004) 0.841 |  |
| VAT | 1.000 (0.996-1.003) 0.862 |  |
| IMAGE | 1.040 (0.985-1.098) 0.160 |  |
| VSR | 0.806 (0.509-1.276) 0.358 |  |

Table S2. Univariate and multivariate Cox proportional hazards regression analysis of RFS.

| Variables | Univariate analysis | Multivariate analysis |
| --- | --- | --- |
|  | HR (95% CI) P | HR (95% CI) P |
| Age | 1.028 (0.999-1.059) 0.058 |  |
| Gender | 1.255 (0.746-2.111) 0.392 |  |
| BMI | 1.170 (0.693-1.975) 0.556 |  |
| Hepatitis B | 1.486 (0.593-3.723) 0.398 |  |
| Liver cirrhosis | 1.957 (0.781-4.901) 0.152 |  |
| Biliary calculus | 0.791 (0.418-1.497) 0.472 |  |
| CEA | 1.000 (0.998-1.002) 0.895 |  |
| AFP | 0.998 (0.991-1.005) 0.585 |  |
| ALT | 1.000 (0.998-1.002) 0.822 |  |
| AST | 1.001 (0.998-1.003) 0.624 |  |
| SMI | 0.984 (0.954-1.015) 0.305 |  |
| SMA | 0.995 (0.986-1.004) 0.292 |  |
| SAT | 0.999 (0.995-1.004) 0.810 |  |
| VAT | 0.999 (0.996-1.003) 0.714 |  |
| IMAGE | 1.035 (0.979-1.094) 0.231 |  |
| VSR | 0.781 (0.495-1.232) 0.288 |  |
